# Microstructure and physical properties of black-aluminum antireflective films†

**DOI:** 10.1039/d4ra00396a

**Published:** 2024-05-10

**Authors:** Cinthia Antunes Corrêa, Joris More-Chevalier, Petr Hruška, Morgane Poupon, Michal Novotný, Peter Minárik, Pavel Hubík, František Lukáč, Ladislav Fekete, Dejan Prokop, Jan Hanuš, Jan Valenta, Přemysl Fitl, Ján Lančok

**Affiliations:** a Institute of Physics of the Czech Academy of Sciences Na Slovance 1999/2 182 00 Prague 8 Czech Republic correa@fzu.cz morechevalier@fzu.cz hruskap@fzu.cz poupon@fzu.cz novotnym@fzu.cz hubik@fzu.cz fekete@fzu.cz prokopdejan@gmail.com lancok@fzu.cz; b Charles University, Faculty of Mathematics and Physics Ke Karlovu 2027/3 121 16 Prague 2 Czech Republic peter.minarik@matfyz.cuni.cz Jan.Hanus@matfyz.cuni.cz jan.valenta@matfyz.cuni.cz; c Institute of Plasma Physics of the Czech Academy of Sciences Za Slovankou 1782/3 182 00 Prague 8 Czech Republic lukac@ipp.cas.cz; d University of Chemistry and Technology, Department of Physics and Measurements Technická 5 166 28 Prague 6 Czech Republic fitlp@vscht.cz

## Abstract

The microstructure and physical properties of reflective and black aluminum were compared for layers of different thicknesses deposited by magnetron sputtering on fused silica substrates. Reflective Al layers followed the Volmer–Weber growth mechanism classically observed for polycrystalline metal films. On the contrary, the extra nitrogen gas used to deposit the black aluminum layers modified the growth mechanism and changed the film morphologies. Nitrogen cumulated in the grain boundaries, favoring the pinning effect and stopping crystallite growth. High defect concentration, especially vacancies, led to strong columnar growth. Properties reported for black aluminum tend to be promising for sensors and emissivity applications.

## Abstract 2

The microstructure and physical properties of reflective and black aluminum were compared for layers of different thicknesses deposited by magnetron sputtering on fused silica substrates.

## Graphical abstract

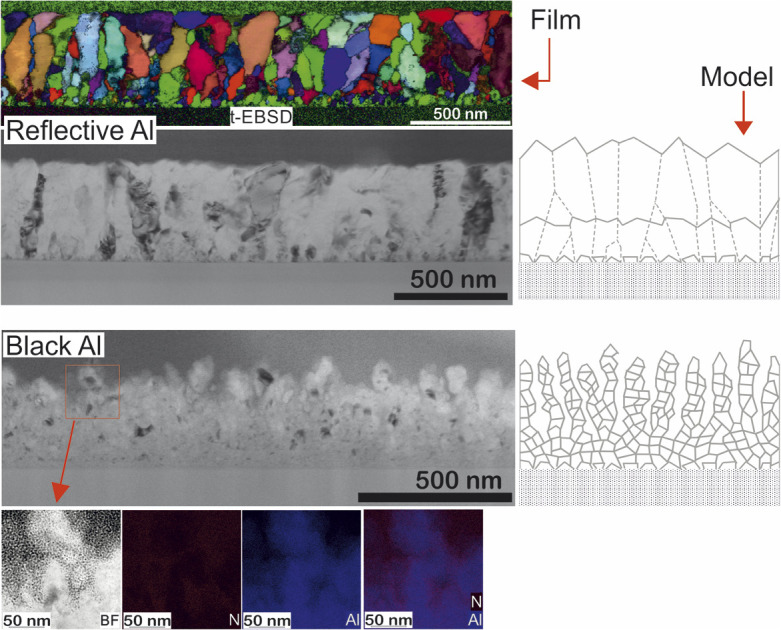

## Introduction

Black metal (BM) absorbers are defined as metals that can strongly trap light due to their plasmonic structures.^1,2^ The black aspect of black metals is due to their subwavelength electromagnetic interactions with light in wide wavebands.^2,3^ The collected light comes from a volume much larger than its physical size, which occurs in localized surface plasmon resonance in multiple wavebands. The photons trapped on the surface transfer their energy into electric and/or thermal energy, allowing for various applications in solar energy harvesting, optical sensing, imaging, electrochemical sensing,^4–9^ antireflection, camouflage, and cloaking.^10–13^

BMs have been prepared by magnetron sputtering,^14–17^ thermal evaporation,^4,18–22^ electrodeposition,^6,7^ deposition on a dielectric substrate with imprinted moth-eye-like nanostructures,^5^ and by laser surface treatment.^10,23–26^ The porosities introduced during the metal films growth or during the treatments before and after the deposition help to create a structure allowing complex subwavelength electromagnetic interactions with the light in wide wavebands.^7^ Black or colored coating has been obtained using metals such as gold, platinum, tungsten, copper, titanium, palladium, or aluminum.^15,22,24,27–32^ Recently, black metals have been utilized to improve the sensitivity of quartz crystal microbalance sensors for gas detection,^9^ and a black aluminum coating was used for converting light into electrical current by pyroelectric effect.^8^

Aluminum is a promising metal for fabricating black metal coatings due to its good adhesion and its low cost, which makes it promising for industrial use. Black aluminum (B-Al) was reported to be prepared by thermal evaporation, while Al film was deposited on an imprinted dielectric substrate.^21,33,34^ An easy and efficient way to fabricate B-Al films has been recently reported using pulsed direct current (DC) magnetron sputtering with a mixture of Ar–N_2_ gases.^16^ Even though positron annihilation lifetime spectroscopy (PALS) showed the presence of pores in the B–Al films^15,17^ and the importance of defects in the film formation,^32^ the growth model remains to be confirmed.

This work presents a comparative analysis of reflective Al (R-Al) and B-Al films deposited with thicknesses varying from 60 nm to 410 nm. Detailed structural analysis is shown for each film, including X-ray diffraction (XRD), atomic force microscopy (AFM), transmission electron microscopy (TEM), and positron annihilation lifetime spectroscopy. Optical properties, resistivity, and Hall effect measurements are also presented. The film growth model and properties are discussed as a function of the thickness of films.

## Experimental

### Film growth

Reflective and black aluminum films were deposited by pulsed DC magnetron sputtering on fused silica substrates. Substrates were fixed on an unheated substrate holder placed at 100 mm from the target. A round-shaped magnetron aluminum target (Al purity of 99.99%) with a diameter of 100 mm was used for sputtering. A DC power supply (Huttinger 3000) combined with a pulse generator (MELEC) was operated at a power of 400 W, with a repetition frequency (*f*) of 10 kHz, a duty cycle of 0.5 μs, and a deposition rate of 80 nm min^−1^. The vacuum chamber was pumped to a base pressure of 2 × 10^−3^ Pa. The magnetron discharge was maintained in a reactive atmosphere of N_2_/Ar (gas purity N6.0 (99.9999%)) with ∼6% of N_2_ for the B-Al films and in a pure Ar atmosphere for the R-Al films. The deposition pressure was kept constant at 0.5 Pa, regulated using a throttle valve at the high-vacuum pump gate. DC voltages were 415 V and 365 V for R-Al and B-Al depositions, respectively. Films were deposited with thicknesses varying from ∼50 nm to ∼440 nm. Thicknesses were estimated by profilometry and electron microscopy and are presented in Table 1.

**Table 1:** Film thicknesses of Al films and B-Al films measured by profilometry

R-Al film thickness (nm)	B-Al film thickness (nm)	Thickness label (nm)
50 ± 10	70 ± 10	60
155 ± 15	200 ± 20	180
240 ± 20	260 ± 20	250
280 ± 20	360 ± 20	320
430 ± 20	400 ± 20	410

### X-ray powder diffraction

Thin films were characterized by X-ray Diffraction using a Bruker Discover diffractometer equipped with a copper anode source (*λ-K*_α_1__ = 1.540 593 Å). The diffractometer is equipped with a detector in Bragg–Brentano geometry. Powder diffraction profiles were fitted using the fundamental approach in the crystallographic software Jana2006.^35^ The parameters used for the fundamental approach were goniometer size 280 mm, receiving slit width 0.1 mm, fixed divergence slit angle 0.5°, filament length 12 mm, sample length 15 mm, receiving slit length 12 mm, both Soller slits 2.5°, and a radiation profile CuK_α_2___analytic. The background was modeled using 10 Legendre polynomials combined with a manual background. The crystal size was refined using Rietveld analysis in the TOPAS V5 code.^36^

### Atomic force microscopy

Atomic force microscopy measurements were carried out at room temperature on an ambient atomic force microscope (Bruker, Dimension Icon) in peak force tapping mode with ScanAsyst air tips (Bruker; *k* = 0.4 N m^−1^; nominal tip radius of 2 nm) or classical tapping mode using Tap 150 Al-G tips (BudgetSensors; *k* = 5 N m^−1^; nominal tip radius <10 nm) for samples with high roughness. The measured topographies had a resolution of 512 × 512 pixels.

### Transmission electron microscopy

The lamellae preparation for TEM investigations was done by FIB in a dual-beam Zeiss Auriga Compact. TEM and t-EBSD images were acquired using a JEOL JEM-2200FS STEM equipped with a field emission gun at 200 kV, a precession device Astar, and a 2k Gatan CCD camera. STEM and EDX images were acquired on a high-resolution TEM Titan Themis at 300 kV, equipped with bright-field and high-angle annular dark field detectors for scanning imaging, with an energy dispersive X-ray analysis super-X spectrometer for EDX.

### Positron annihilation spectroscopy

Positron annihilation lifetime spectroscopy experiments were carried out on a pulsed mono-energetic positron beam MePS (Mono-energetic Positron Spectroscopy)^37^ operating at the ELBE facility^38^ in the Helmholtz-Zentrum Dresden-Rossendorf. The energy of incident positrons in the beam varied from 1 to 16 keV, corresponding to the mean positron implantation depth into Al from 15 nm to 1300 nm, as calculated using the Makhovian implantation profile.^39^ Positron lifetime spectra were collected using a digital spectrometer with a time resolution of 250 ps (FWHM of the resolution function). A total statistics of 10^7^ annihilation events was collected for each spectrum. The decomposition of positron lifetime spectra into individual exponential components was performed using the PLRF code.^40^

### Reflectivity

Optical properties were characterized by spectrophotometric reflectance measurements. Specular reflectance was measured on a spectrophotometer (PerkinElmer, UV/VIS/NIR Lambda 750) equipped with Universal Reflectance Accessory. A constant angle of incidence of 8° and the range of wavelengths of 190 nm–1200 nm were used. Diffuse reflectance measurements were performed using a spectrophotometer (Analytik Jena, Specord 250) equipped with an integrating sphere for wavelengths ranging from 320 nm to 1100 nm. A Spectralon (LabSphere) reflectance standard was used as a 100% reflectance reference.

### Electrical measurements

Resistivity and Hall effect measurements were carried out using the differential van der Pauw (vdP) method in a quasi-square arrangement at room temperature (298 ± 1) K, using a Keithley 6221 current source, and two electrometers, a Keithley 6514 with a Keithley 2182A nanovoltmeter, which recorded the voltage difference between the electrometers together with a Keithley 708B switching matrix. Samples were contacted to the electrical measurement systems using Cu tips. The linearity of the contacts was checked for all measured samples to ensure contact ohmicity. A magnetic field of 0.20 T was applied for the Hall effect measurements.

## Results and discussion

### Film crystallization and surface morphology

Deposited films (see Fig. A1 in the ESI†) had thicknesses from ∼50 nm to ∼440 nm. To simplify the discussion, hereinafter, we will use film labels 60 nm, 180 nm, 250 nm, 320 nm, and 410 nm.

**Fig. 1:**
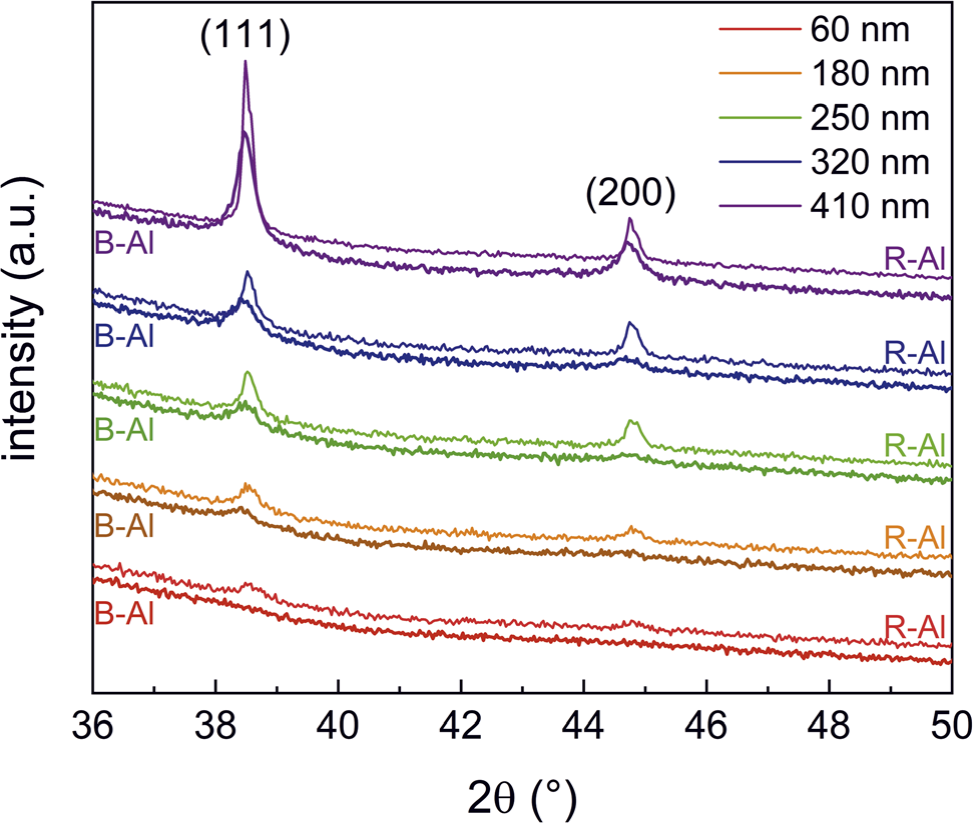
XRD patterns of R-Al and B-Al films as a function of film thicknesses.

XRD 2*θ* scans as a function of the thickness of R-Al and B-Al layers are presented in Fig. 1 for 2*θ* from 36° to 48°. The polycrystallinity of the films is observed through four diffraction peaks, with positions at 2*θ* ≈ 38.5°, 44.8°, 65.8°, and 78.2°. These peaks correspond to the planes (111), (200), (220), and (311) of the fcc aluminum structure, which has a cubic lattice parameter *a* ≈ 4.05 Å and space group *Fm*3̄*m*. B-Al layers present lower intensities and broader peaks than those from the R-Al layers.

Note that no diffraction peak was observed for the thinnest B-Al film, indicating that the mean crystallite size of this film is too small to offer a coherent diffraction pattern. The Rietveld refinement was performed using the crystallographic information file (cif) of Al^41^ from the ISCD database.^42^ Only the lattice parameter *a* and mean crystallite sizes were refined. Fitted results are presented in Fig. 1.

After several tests using the fundamental approach, it has been observed that the refinement of crystallite strain can be neglected for such small crystallites.

Concerning the R-Al films, a relative difference can be observed between the aluminum bulk lattice parameter and the film parameters in a range of 0.01–0.06%, corresponding to a slight compressive strain of the cell. The mean crystallite size in R-Al films increases with the film thickness from ∼19 nm for the 60 nm thick film to ∼74 nm for the 410 nm thick one. These results are coherent with the growth of polycrystalline Al.^16,43,44^ On the other hand, tensile strains are observed with higher relative differences between the bulk and the B-Al film lattice parameters: 0.18–0.41% for thin B-Al films (thickness ≤ 250 nm) and 0.01–0.05% for thick B-Al films (thickness ≥ 320 nm). The mean crystallite size in B-Al films saturates with the increase of the film thickness from ∼11 nm for the 180 nm thick film to ∼26 nm for the 410 nm one. This result agrees with the behavior of domain growth as a function of nitrogen concentration. The presence of impurities during the growth of metal films tends to affect the lattice parameter and the domain size.^45^ When the concentration of impurities is low, the domain (or grain) size remains constant, and stress is induced in the lattice parameter, corresponding to the regime I discussed by Yu and Thompson.^45,46^ When the presence of impurities becomes higher, the domain size is affected and becomes a function of the ratio between the impurity and metal impingement fluxes, corresponding to regime II of the metal film growth. The increase in the mean crystallite size observed for R-Al films is coherent with the one Dulmaa *et al.*^47^ reported. They reported equivalent crystallite size variation for Al as a function of the film thickness, namely from ∼20 nm for a thickness of ∼30 nm to ∼90 nm for a thickness of ∼1000 nm. The slight difference with our work is certainly correlated with the lower flux ratio between impurities and metal. The influence of the impurity concentration was well reported by Dulmaa *et al.*,^47^ and it explains the substantial decrease in the mean crystallite size as a function of the B-Al film thickness reported here (Table 2). This phenomenon is correlated with the decrease in the atom mobility energy during the deposition growth.^43,47^

**Table 2:** Evolution of the unit cell parameter *a* (Å) and the crystallite size of R-Al and B-Al films obtained by Rietveld refinement of the XRD data

Thickness (nm)	Reflective aluminum		Black aluminum	
	*a* (Å)	Crystallite size (nm)	*a* (Å)	Crystallite size (nm)
≈60	4.048(7)	19(4)	—	—
≈180	4.050(3)	25(3)	4.066(3)	11(2)
≈250	4.047(2)	37(3)	4.057(2)	14(2)
≈320	4.048(1)	40(3)	4.050(3)	19(2)
≈410	4.0486(6)	74(5)	4.0515(9)	26(1)
Bulk^41^	4.0495(8)		4.0495(8)	

**Fig. 2:**
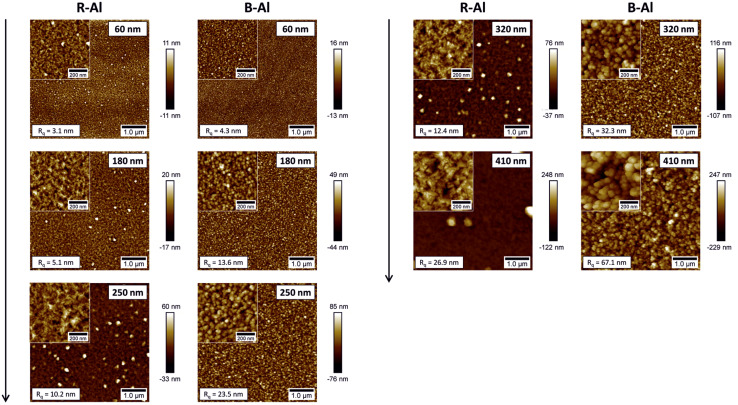
AFM images of R-Al and B-Al layers as a function of film thicknesses.

Fig. 2 shows the surface morphology of R-Al and B-Al films obtained by AFM, where *R*_q_ is the root mean square (rms) roughness. R-Al layers present dense surfaces with coalescing grains and emerging second-generation well-defined crystals, which become dominant for film thicknesses above 1 μm. As the thickness of R-Al films increases, the mean crystallite (observed by XRD) and grain (observed by AFM) sizes also increase, following the V-growth mode, similar to that described elsewhere.^16,17^ This is also supported by increasing *R*_q_ from 3.1 nm to 26.9 nm. Substantial development of grain morphology is observed for B-Al layers. Surfaces are composed of multiple small spherical grains that seem weakly densified compared to R-Al films and form cauliflower-like structures. A significant roughness increase from 4.3 nm to 67.1 nm is observed with the increasing thicknesses. Note that a precise determination of the roughness of B–Al films by AFM is limited by the dimensions of the tip, and therefore, their roughness is presumably even higher.

### Transmission electron microscopy

**Fig. 3:**
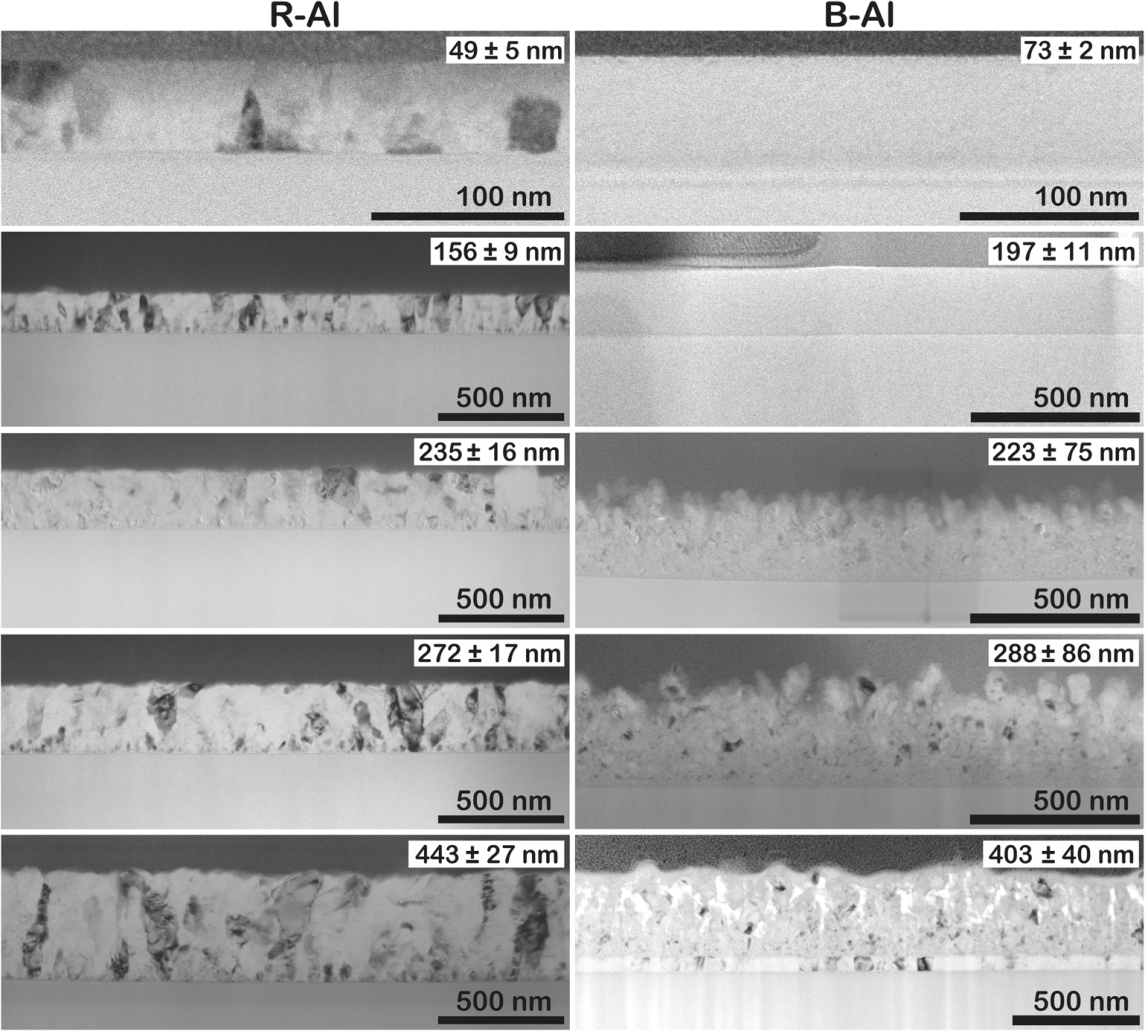
TEM images of the R-Al and B-Al films with increasing thickness. The thickness of the film is shown in the top right corner.

Fig. 3 shows the cross-section of the films in the low-magnification Bright field (BF) TEM images. The thicknesses of the layers are shown in the inset in the top right corner of each thin film figure. R-Al films present a small, randomly oriented island on the surface of the substrate, most likely due to the deposition on an unheated substrate. The polycrystalline growth can be better observed in Fig. A2 (ESI†), where the transmission electron backscatter diffraction (t-EBSD) image is presented with the orientation of the Al crystals in the R-Al film with a thickness of 410 nm. The t-EBSD image confirms that there is no preferred orientation of the grains within the film. An electron diffraction pattern (Fig. A3 in the ESI†) was acquired on the 410 nm thick B-Al film and indexed using the Al unit cell parameter *a* = 4.054 Å. The observed pattern shows diffraction rings of mutually misoriented Al crystallites, characteristic of a polycrystalline sample, instead of the well-defined diffraction peaks, characteristic of single crystals.

**Fig. 4:**
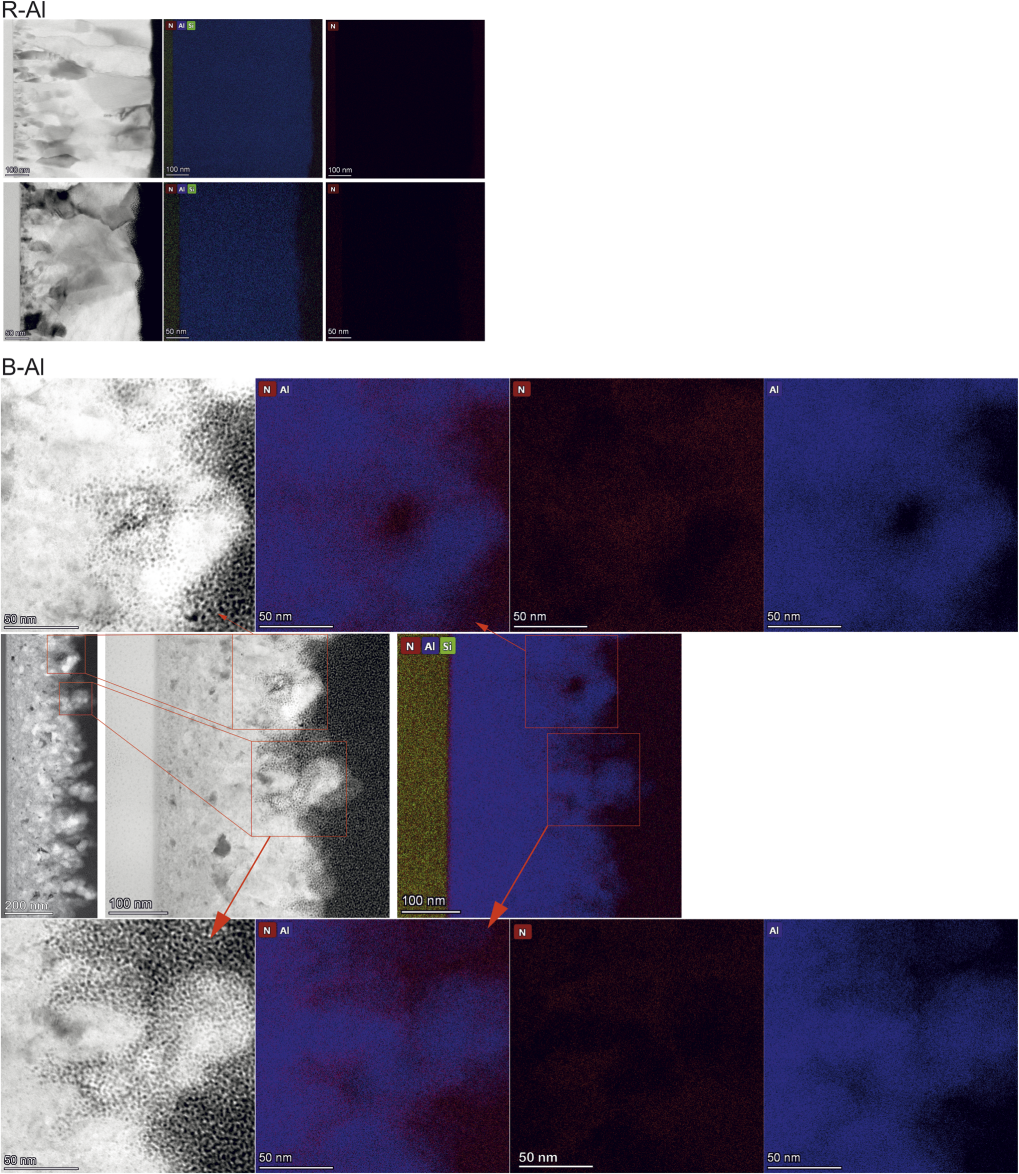
EDX maps of the 410 nm and 320 nm thick R-Al, and 320 nm thick B-Al films. While the R-Al presents a high Al concentration in the film and a very low concentration of N, B-Al shows increasing porosity as the film grows thicker, and nitrogen can be observed within the Al grains. More detailed N content within the pores is shown in a higher magnification image in the inset maps for two positions.

Concerning the B-Al layers in Fig. 3, two regions can be observed with the increase of thicknesses. Above the layer of small islands, bigger crystals are present, coming from the coalescence of smaller ones. The last region is formed by column-like crystals (see Fig. A4 in the ESI†), following the film growth behavior described elsewhere.^16,48^ Even though one cannot accurately define the small island sizes, a qualitative comparison with the column-like crystals gives a clear image of the different regions in the layer formation. B-Al films have increasing porosity as a function of the thickness of the film. In this case, surface roughness caused by N incorporation decreases the surface diffusion of Al, generating porosities. The nitrogen incorporation does not significantly change the film growth of the thinnest B-Al layer (60 nm), which has similar porosity as the R-Al layer with the same thickness. The incorporation becomes significant as thickness increases, generating defects such as voids, which strongly reduce the adatom diffusion in the crystallite boundaries.^16,17,22^ This influence can be seen in Fig. 4, which presents the EDX images of the 410 nm and 320 nm thick R-Al films, and that of the 320 nm thick B-Al. The R-Al films have a dense concentration of Al with a very low concentration of N impurities, while the porous B-Al film contains a higher amount of N and lower N content in the regions where the Al grains are present. This high concentration of nitrogen in the boundaries favors AlN amorphous phase, which may tend to crystallize for a thicker layer over 1 μm thick, as previously reported.^17^ Fig. A5 (ESI†) presents three depth profiles around the column shown at the bottom of Fig. 4. The three depth profiles show the evolution of the distribution of elements. The first profile shows the top edge of the column with a higher Al concentration at the Al crystal and decreased Al concentration from around 75 nm until the Pt protection layer, slightly increasing when the profile line approaches the big Al crystal at ∼140 nm. The N content oscillates with a slightly higher concentration around the pores and a lower concentration close to the Al crystals. The oxygen concentration detected in the film comes from the Al oxidation under the air, which has been increased during the lamella preparation and storage under ambient conditions. The second and third profiles show the same behavior, with an increased N concentration at the boundaries, where the Al concentration decreases, and a decreased N concentration where the Al concentration increases, corresponding to the next Al crystallite. A clear link can be made between voids and porosity formation in the B-Al films and the nitrogen concentration incorporated during the deposition. An increase of the N in the porosities indicates that the nitrogen accumulation tends to stop the grain growth by increasing the pinning effect, favoring the creation of defects. Bigger pores increases the area where N can diffuse, increasing the porosity even more. Therefore, the images confirm that the N impurities favor the pinning effect during the B-Al film growth.^16,49^ Crystallite growth is affected by the concentration of impurities, which induces a strong pinning effect on the grain boundaries. The adatom diffusion is reduced in the boundaries, and the sputtered atoms tend to fix on the minimum surface energy, favoring a strong columnar growth and the formation of defects like porosities.^34,46^ The film aims for the most stable thermodynamic situation, bringing the columns to be oriented with the plane with minimal surface tension energy parallel to the substrate.^45–49^ The resulting nano and microstructure after deposition is similar to a moth-eye structure.

### Positron annihilation

**Fig. 5:**
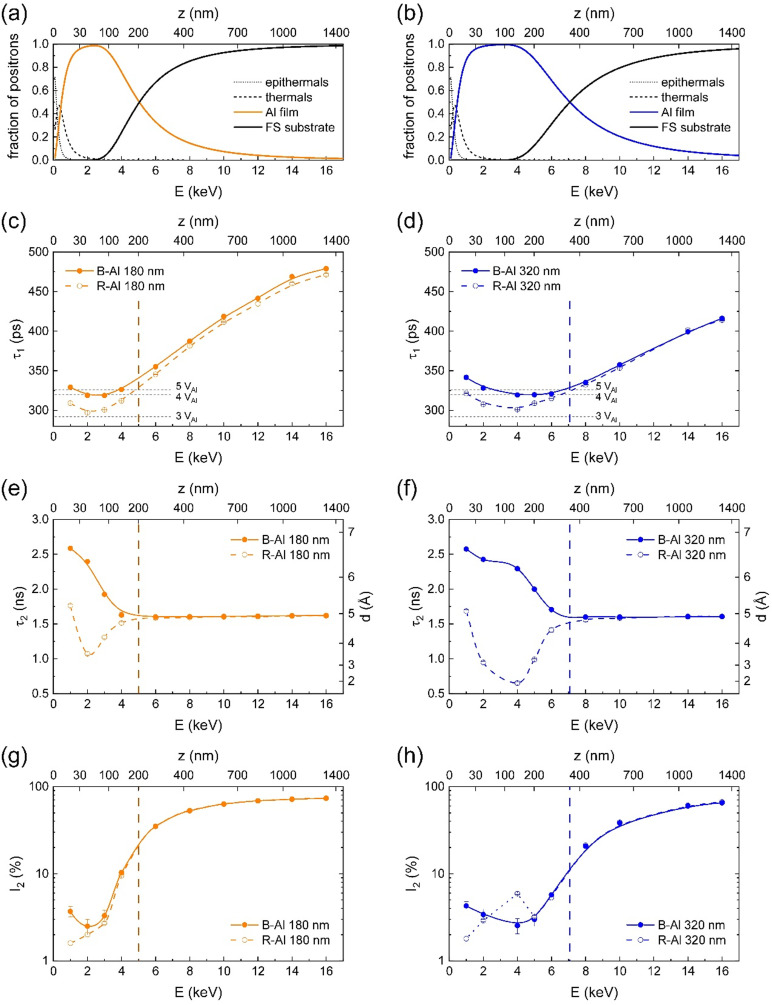
PALS results as a function of positron energy *E* for 180 nm (in orange, left) and 320 nm (in blue, right) B-Al films (full points) and R-Al films (open points): (a and b) positron fractions calculated by VEPFIT; (c and d) lifetime of positrons annihilated as particles; (e and f) lifetime of o-Ps pick-off annihilations; (g and h) intensity of Ps component. The mean positron penetration depth *z* is depicted at the top axis for each plot. The vertical dashed lines mark the Al film – FS substrate boundary.

The defect structure was characterized by PALS.^15^ The results for B-Al and R-Al films with thicknesses of 180 nm and 320 nm are shown in Fig. 5. Mean positron penetration depth *z*, corresponding to the energy *E* of incident positrons, is depicted on the top axis for each plot. To properly describe the depth resolution provided by the positron beam, fractions of positron annihilating (i) at the surface (epithermal and thermal positrons), (ii) in the Al film, and (iii) in the fused silica (FS) substrate were modeled using the VEPFIT program^50^ and are shown in Fig. 5(a) and (b). One can see in the figures that the boundaries between the layers (a)–(c) are blurred due to the broadening of the implantation profile with increasing positron energy and positron diffusion. The measurements at 1 keV and 2 keV describe the situation when most of the positrons annihilated in the Al layer while a part of the positrons still annihilated at the surface, which is characterized by a different lifetime. A low-energy region, *E* < 5 keV for 180 nm thick films and *E* < 7 keV for 320 nm thick films, describes annihilations predominantly in the Al film. The Al film – FS substrate boundary is marked with a vertical dashed line and is defined by the equal fraction of positrons annihilating in the film and the substrate.

Positron lifetime spectra were decomposed into two exponential components: (i) short-lifetime component *τ*_1_ representing positrons annihilated as particles, and (ii) long-lifetime component *τ*_2_ as a contribution of pick-off annihilations of *ortho*-positronium (*o*-Ps). The *para*-positronium (*p*-Ps) contribution with a lifetime fixed at 125 ps was included in the model function. Intensities of *o*-Ps and *p*-Ps components were constrained to the ratio 3 : 1, and their sum was marked as *I*_2_. The evolution of components *τ*_1_ with energy *E* is shown in Fig. 5(c) and (d); analogously, components *τ*_2_ as a function of energy *E* are shown in Fig. 5(e) and (f). Intensities of positronium component *I*_2_ complement positron intensities *I*_1_ and are included in Fig. 5(g) and (h).

At the low-energy region (on the left to the vertical dashed lines), characterizing the Al film, the component *τ*_1_ exceeds significantly the Al bulk lifetime of 164 ps as well as the lifetime of 243 ps for positrons trapped in Al monovacancies,^51,52^ indicating positrons are trapped in open-volume defects with a size comparable to small vacancy clusters. Considering the nanocrystalline structure of B-Al and R-Al films, as described in previous sections, and the relatively short positron diffusion length compared with the mean grain size, vacancy clusters are most likely located at the grain boundaries but also partially inside the Al grains. The average size of vacancy clusters can be estimated by comparison of lifetimes *τ*_1_ with those obtained from *ab initio* theoretical calculations^52^ to 3 or 4 vacancies for R-Al films and 4 vacancies for B-Al films. Therefore, B-Al films exhibit a more open structure at the atomic level compared to the more compact structure of R-Al films.^17^ At higher energies (on the right to the vertical dashed lines), lifetimes *τ*_1_ for B-Al and R-Al follow the same curve, representing positrons annihilating predominantly in the FS substrate.

In R-Al films, Ps can be formed either in the natural Al_2_O_3_ layer at the surface or in the FS substrate. In B-Al films, Ps can also be formed in nanocavities between the column-like grains.^17^ The presence of Ps in B-Al is proved by an enhanced lifetime *τ*_2_ in the low-energy region (Fig. 5(e) and (f)). Employing the Tao-Eldrup-Ito model,^53^ which describes spherical cavities, we can estimate their mean diameter as 6 Å.

The Ps intensity in B-Al is below 5%, *i.e.*, vacancy clusters are the dominant type of defect in both types of films. However, in the Al lattice, the dimension of non-relaxed vacancy clusters with an average size of 3 or 4 vacancies is approximately 5 Å, close to the mean diameter of cavities calculated from *o*-Ps lifetime. In some cases, the formation of Ps in open volumes at the grain boundaries with locally enhanced nitrogen content may be favorable compared to positron trapping. Alternatively, the cavities can be connected in chains, eventually forming larger nanopores along grain boundaries, which are observable by TEM.

In the amorphous FS substrate, the pick-off annihilation of *o*-Ps is a dominant process, characterized by the intensity *I*_2_, gradually increasing with the increasing fraction of positron annihilating in FS and reaching 70–80%. The mean lifetime *τ*_*2*_ agrees well with the lifetime of 1.50(5) ns obtained by the digital spectrometer.^54^ However, its evolution near the film–substrate boundary is significantly more rapid than the evolution of component *τ*_1_, likely due to the non-uniform distribution of cavities, which develop with the increasing film thickness, as observed by TEM.

### Electrical and optical properties

**Fig. 6:**
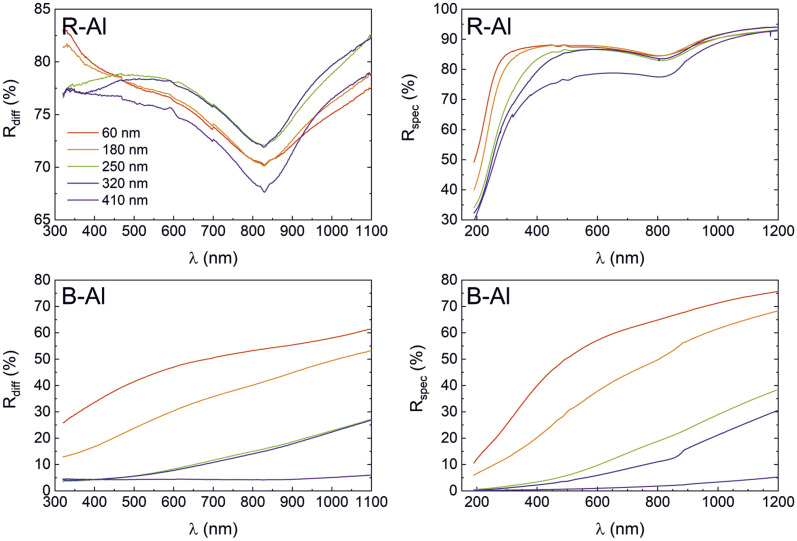
(Color online) Diffuse *R*_diff_ and specular *R*_spec_ reflectances of R-Al films (top) and of B-Al films (bottom), as a function of their thicknesses: 60 nm (in red), 180 nm (in orange), 250 nm (in green), 320 nm (in blue), and 410 nm (in purple).

Specular (*R*_spec_) and diffuse (*R*_diff_) reflectances of R-Al and B-Al films as a function of their thicknesses are presented in Fig. 6. The diffuse reflectance variation of R-Al films shown in Fig. 6(a) is relatively stable as the thickness increases. Equivalent results around 75% are observed for the 60 nm and 180 nm thick films. Then, the *R*_diff_ for the 250 nm and 320 nm thick films increases to ∼78%. Finally, *R*_diff_ decreases to ∼73% for the 410 nm thick film. This value is very similar to those reported in our previous work,^16,17^ which were *R*_diff_ ∼77.5% and ∼71% for ∼500 nm and ∼1590 nm thick B-Al films, respectively. On the other hand, a decrease in the specular reflectance of R-Al film is noted in Fig. 6(b) as a function of their thicknesses. This phenomenon is more marked for the applied photons with wavelengths shorter than 600 nm. The behavior correlates with the increase of the surface roughness linked to the increase in film thicknesses from 3.1 nm to 26.9 nm. Similar results were reported for Al films with thicknesses varying from ∼20 nm thick to ∼550 nm, which were also attributed mainly to the increase in surface roughness.^55^ The same phenomenon was reported for an R-Al layer of 1.5 μm where the high roughness increased the light scattering, which tended to obtain a lower specular reflectance than a diffuse reflectance.^17^ The characteristic absorption peaks at ∼825 nm observed in all R-Al spectra correspond to the interband transitions at the *W* point of the Brillouin zone in the Al band structure.^56,57^

The diffuse and specular reflectances of B-Al samples as a function of the film thicknesses are reported in Fig. 6(c) and (d). A decrease in both reflectances is observed with the increase in the film thicknesses. First, the *R*_spec_ intensity is more intense than the *R*_diff_ for the thin B-Al layers of 60 nm and 180 nm. Then, an inversion of the reflectance intensities is observed with the thickness increasing from 250 nm to 410 nm to finally obtain a *R*_diff_ intensity higher than *R*_spec_ intensity for the B-Al film of 410 nm. The reflectance intensity reduction is connected to the increase of the roughness as well as the porosity concentration, which is in line with the AFM and the PALS results presented in previous sections. Light absorption is a multi-step process where the surface morphology and the localized surface plasmon resonance (LSPR) play essential roles.^7,16^ The extension of mutually isolated plasmonic structures was proposed by the confinement to the sub-20 nm level due to nanostructures with complex morphologies and size distributions.^7^ In the case of B-Al, the average size of the Al particles of 21 nm separated by AlN in grain boundaries and the presence of nanopores, allow the LSPR phenomenon.

**Fig. 7:**
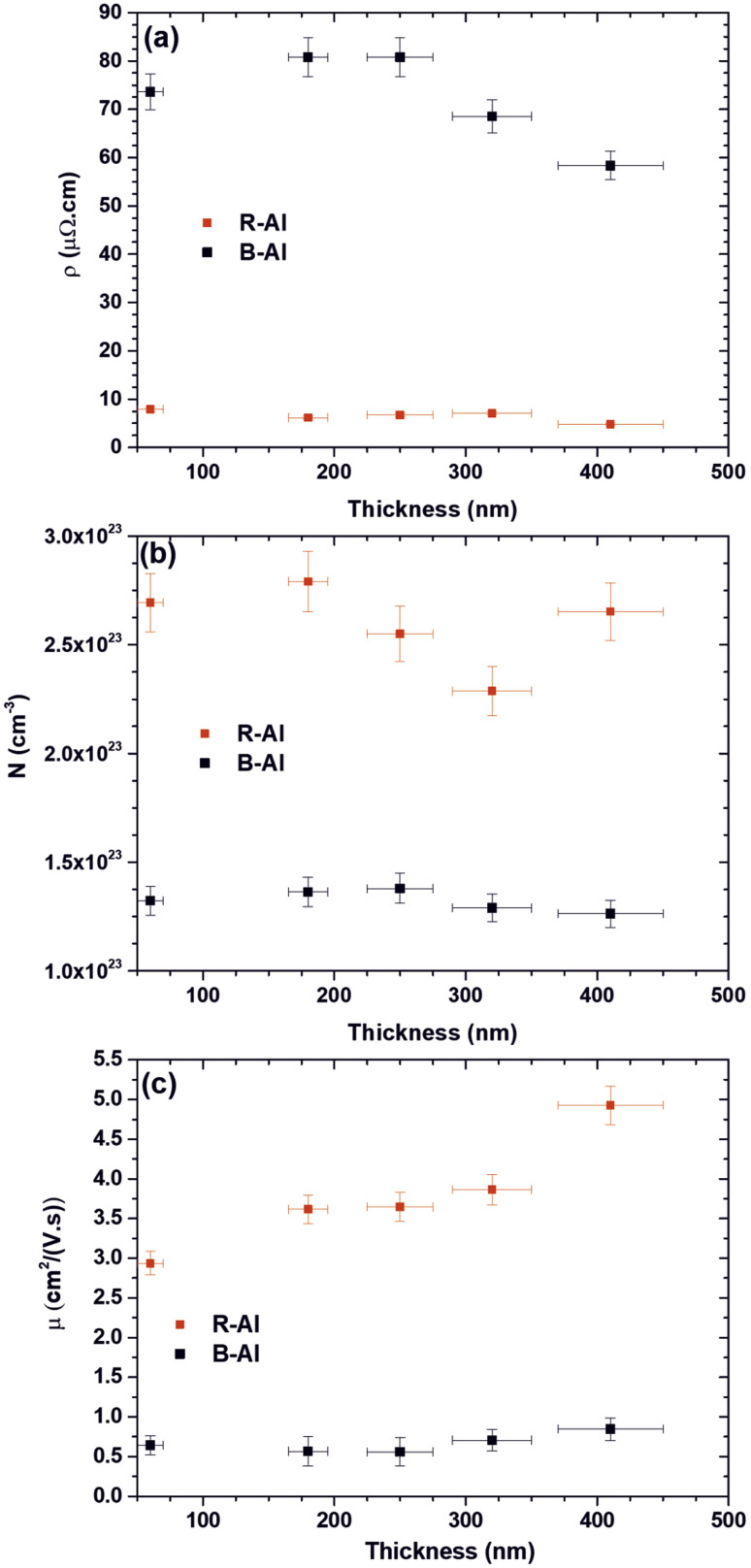
The electrical properties of R-Al and B-Al films are a function of the film thicknesses. In (a) is presented the resistivity, in (b) the carrier concentration, and (c) the carrier mobility, respectively.

The electrical properties of R-Al films and B-Al films as a function of the thickness of films are presented in Fig. 7. The resistivities of the R-Al and B-Al layers are shown in Fig. 7(a). The reported values slightly vary from 7.9 μΩ cm to 4.8 μΩ cm with the increase in layer thickness of the R–Al films. A similar effect of the decreased resistivity in the Al films with the increase of the thickness has already been reported for ultrathin films (thickness lower than 100 nm)^58–60^ as well as for thicker films^58,61^ where both surface scattering and grain boundary scattering play an important role. The resistivity value obtained for the thickest layer is close to these reported in Al films of ∼3 μΩ cm which is almost the bulk aluminum metal.^61–63^ Concerning the evolution of B-Al resistivity as a function of the increase of the film thickness, the value increases first from 73.6 μΩ cm for the B-Al layer of 60 nm, to 80.8 μΩ cm for the B-Al film of 250 nm.

Then, the B-Al resistivity decreases at 68.5 μΩ cm for the film of 320 nm and 58.3 μΩ cm for the film of 410 nm. The higher resistivity value measured for the B-Al films is correlated with the larger number of grain boundaries than in the equivalent Al films.^64^ The slight increase of the resistivity reported for the smallest thicknesses of 60 nm and 180 nm can be correlated with a slight increase in the grain boundaries between samples. Then, the decrease reported for the B-Al films of 320 nm and 410 nm can be connected to the slight increase in the crystal size in the films, reducing the number of grain boundaries. Fig. 7(b) shows the carrier concentration in R-Al and B-Al samples as a function of the film thickness. The R-Al films present globally a twice higher carrier concentration of ∼2.6 × 10^23^ cm^−3^ than the B-Al films of ∼1.3 × 10^23^ cm^−3^. An erratic change is reported with the thickness increase of the R-Al films with, at first, a slight increase from 2.69 × 10^23^ cm^−3^ to 2.79 × 10^23^ cm^−3^ for the 60 nm and 180 nm films. Then, a decrease followed by an increase is observed from 2.29 × 10^23^ to 2.65 × 10^23^ for the 320 nm and 410 nm. The carrier concentration is strongly connected to the impurity concentration in the films, especially in semiconductors.^65–67^ In this work, we assume that the erratic variation of the carrier concentration is mainly connected to the concentration of impurities, especially carbon and oxygen, introduced during the deposition step. This variation in the concentration of impurities is induced by the variation of the based pressure before deposition, which is around 2 ± 1 × 10^−3^ Pa. The same conclusion is assumed concerning the variation observed for the B-Al carrier concentration as a function of the thickness of films.

The electron carrier mobilities of R-Al films and B-Al films as a function of the thickness are presented in Fig. 7(c). The R-Al carrier mobilities increase with the thicknesses from 2.94 cm^2^ V^−1^ s^−1^ at 60 nm to 4.92 cm^2^ V^−1^ s^−1^ at 410 nm. This variation correlates with the growth of crystals increasing with the time deposition.^16,43,44^ For depositions performed in the same pressure and temperature conditions, the microstructural evolution during the film deposition step tends to increase the coalescence of islands and the coarsening of grains with the increase of the time deposition, which tends to the development of a continuous structure. This development reduces the number of boundaries with the grains coarsening, increasing carrier mobility. The carrier mobilities of B-Al films are lower than in R-Al films. They vary slightly from 0.64 cm^2^ V^−1^ s^−1^ for the film at 60 nm to 0.85 cm^2^ V^−1^ s^−1^ at 410 nm. The larger number of grain boundaries in the B-Al films due to the N_2_ gas used during the deposition step tends to stabilize the crystal size, as reported by the XRD analysis in Table 2. This limitation increases resistivity and reduces carrier mobility. The slight increase in the mobility observed for the thickest B-Al film of 410 nm is correlated with the slight crystal size increase reported in Table 2.

## Conclusions

The growth of reflective and black aluminum films was studied through layer thicknesses varying from 60 nm to 410 nm deposited on fused silica substrates. The reflective polycrystalline aluminum layers presented the Volmer–Weber growth mechanism. An increase in crystallite and grain sizes was observed as a function of the layer thickness, leading to the presence of small crystallites/grains on the bottom part of the film and larger crystallites/grains on the top of the film. The expected optical and electrical properties of reflective Al were reported for the thickest deposited layers of 410 nm. However, the presence of nitrogen (∼6-7%) in the aluminum deposition changed the Volmer–Weber growth mechanism. The crystalline growth saturated quickly at around ∼20 nm despite the increase in the film thickness. The nitrogen accumulation in the grain boundaries favored the pinning effect and generated a high concentration of defects like porosities and vacancy clusters of 5 Å in the B-Al films. This occurred because of a weak coalescence and strong columnar growth. The generated layer structure favored the light trapping and the extremely low measured reported reflectance (diffuse and specular). The large concentration of grain boundaries increased the electrical resistivity at ∼60 μΩ cm for the B-Al layer of 410 nm in comparison to ∼5 μΩ cm for the R-Al layer of 410 nm. This higher resistivity correlated with the lower carrier concentration and mobility measured on the black aluminum layers instead of the reflective ones. These properties can favor applications in optics, emissivity, and sensors.

## Author contributions

The manuscript was written through the contributions of all authors. All authors have approved the final version of the manuscript. Cinthia Antunes Corrêa: data curation, formal analysis, investigation, validation, writing – original draft, writing – review & editing. Joris More-Chevalier: conceptualization, data curation, formal analysis, investigation, methodology, validation, visualization, writing – original draft, writing – review & editing. Petr Hruška: conceptualization, data curation, formal analysis, investigation, validation, visualization, writing – original draft, writing – review & editing. Morgane Poupon: data curation, formal analysis, resources, validation, writing – original draft, writing – review & editing. Michal Novotný: project administration, funding acquisition, validation, visualization, writing – original draft, writing – review & editing. Peter Minárik: formal analysis, validation, writing – original draft. Pavel Hubík: data curation, formal analysis, validation, writing – original draft, writing – review & editing. František Lukáč: data curation, formal analysis. Ladislav Fekete: data curation. Dejan Prokop: data curation, formal analysis. Jan Hanuš: formal analysis, validation, writing – original draft. Jan Valenta: data curation, validation, writing – original draft. Přemysl Fitl: formal analysis, funding acquisition, resources. Ján Lančok: project administration, funding acquisition, resources.

## Data availability

The main data presented in this study are available at https://doi.org/10.57680/asep.0585608.

## Conflicts of interest

There are no conflicts to declare.

## Supplementary Material

RA-014-D4RA00396A-s001
