# Supplementary material for: Microstructure and physical properties of black-aluminum antireflective films
Source: RSC Adv. 2024 May 10;14(22):15220–31. doi: 10.1039/d4ra00396a (PMC11082876; doi:10.1039/d4ra00396a)
Supplement: RA-014-D4RA00396A-s001 [file RA-014-D4RA00396A-s001.pdf]

Electronic Supplementary Material (ESI) for RSC Advances.
This journal is © The Royal Society of Chemistry 2024

ARTICLE

Microstructure and physical properties of Black-Aluminum antireflective films

Cinthia Antunes Corrêa*[a,b], Joris More-Chevalier[a*], Petr Hruška[a,b], Morgane Poupon[a], Michal Novotný[a], Peter Minárik[b], Pavel Hubík[a], František Lukáč[c], Ladislav Fekete[a], Dejan Prokop[a,b], Jan Hanuš[b], Jan Valenta[b], Přemysl Fitl [a,d] and Ján Lančok[a]

[a]Institute of Physics of the Czech Academy of Sciences, Na Slovance 1999/2, 182 21 Prague 8, Czech Republic
[b]Charles University, Faculty of Mathematics and Physics, Ke Karlovu 2027/3, 121 16 Prague 2, Czech Republic
[c]Institute of Plasma Physics of the Czech Academy of Sciences, Za Slovankou 1782/3, 182 00 Prague 8, Czech Republic
[d]University of Chemistry and Technology, Department of Physics and Measurements, Technická 5, 166 28 Prague 6, Czech Republic

Main data presented in this study are available at https://doi.org/10.57680/asep.0585608

Supplementary information

Films' appearance

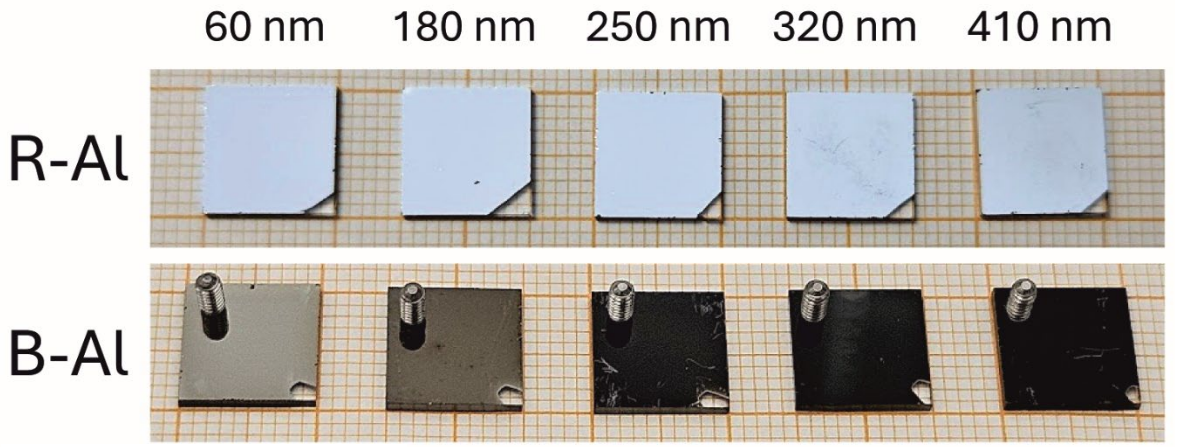

Figure A1: R-Al and B-Al films and their appearance.

Figure A1 presents the R-Al and B-Al films as grown.

Transmission electron microscopy

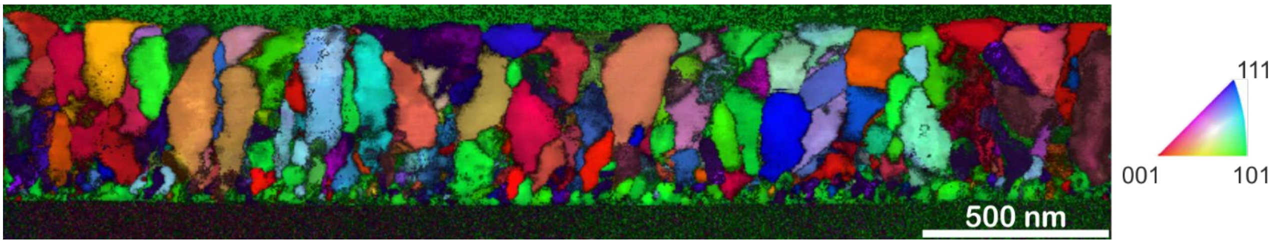

Figure A2: Astar orientation map of the R-Al film with a thickness of ~410 nm.

Please do not adjust margins

Figure A2 presents the t-EBSD of the 410 nm thick R-Al film, showing that there is no preferred grain orientation of the Al crystals during the film growth. Three regions can be observed in the film: the first one, in contact with the substrate (at the bottom of the figure), contains small crystalline grains; the second one has bigger crystals formed by the coalescence of the smaller ones; the third region has crystalline columns with no preferred orientation. Grain orientation can be identified using the color-coded inverse pole figure shown on the right side of the figure.

Figure A3 shows diffraction rings observed by electron diffraction of the 410 nm thick B-Al film. The pattern was indexed using the tool ringGUI from CrysTBox[1] and the Al unit cell parameter a = 4.054 Å[2], according to the crystallographic information file obtained in the Inorganic Crystal Structure Database[3].

Figure A4 presents the column-like structure of the 410 nm B-Al film. The red line indicates where the EDX line scan mapping was performed on the cross-section of the lamella. The graph on the right side shows the higher Si content on the fused silica substrate (~ 100 nm), from where the B-Al film starts. Al content is high at the Al grains, with increasing N content when the line crosses the pore at ~220 nm and again close to the top of the film at ~370 – 420 nm. After that, the Pt protection layer is present.

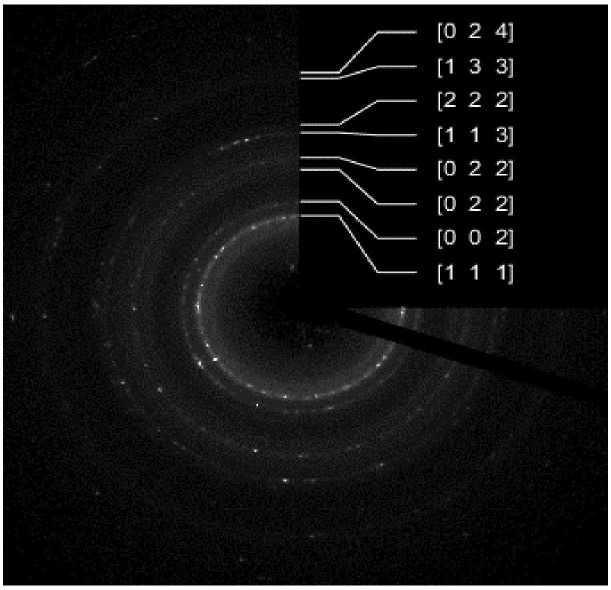

Figure A3: Electron diffraction of the B-Al film (~410 nm thick).

Figure A5 presents three line profiles of one region of the 250 nm B-Al film, shown in Figure 4 in the manuscript. The three line profiles start with around 70% of Al concentration, where the Al crystal is present.

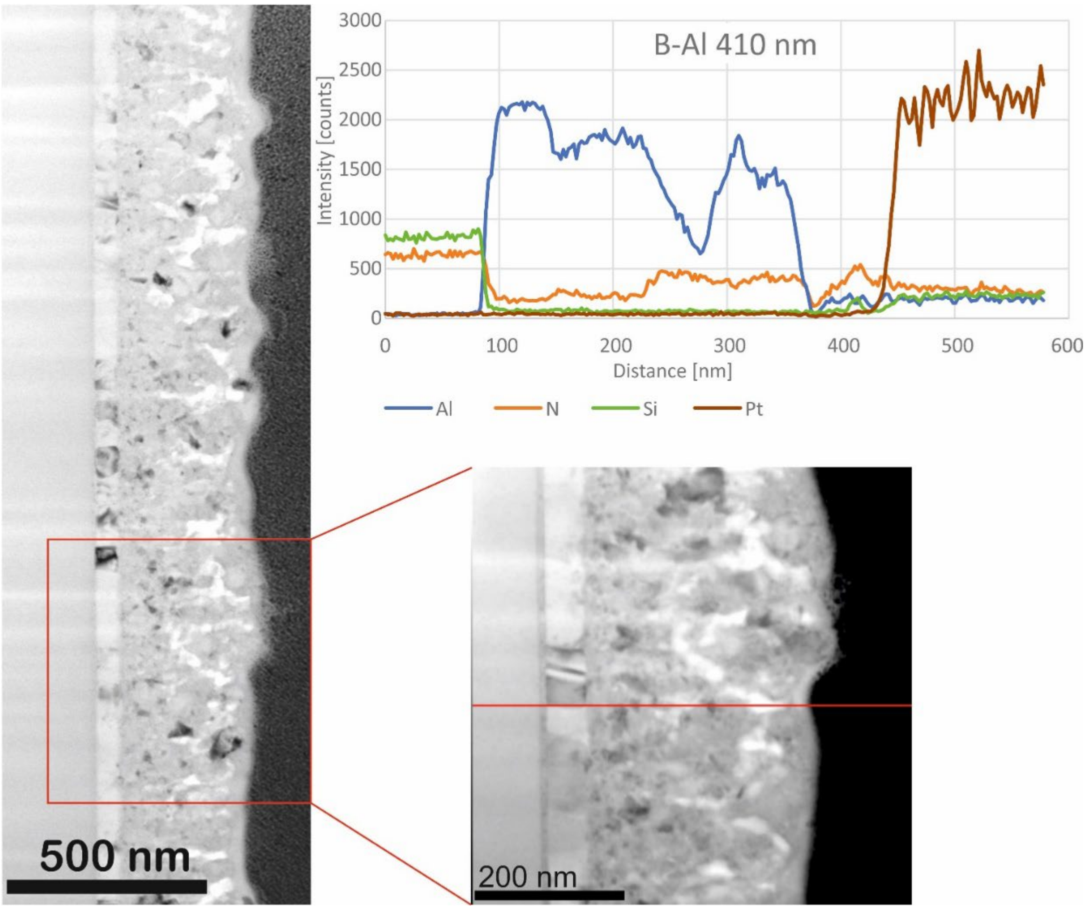

Figure A4: Cross section of the 410 nm B-Al film, and the line profile along the film thicknesses, as shown by the red line in the inset at higher magnification. The graph on the upper right corner shows the Al, N, Si and Pt contents on the film along the line profile.

 This journal is © The Royal Society of Chemistry 20xx

Please do not adjust margins

The first line profile shows the top edge of the column with decreasing Al concentration at ~25 nm, where a small pore is present. A slight increase of the Al content is observed around 60-75 nm, where an Al grain is located, and again around 140 nm, when the line profile approaches the big grain on the right side of the image. N content has a slight increase from below 20% to almost 30% at around 25 nm, where the small pore is present. Another slight increase starts at 50 nm until ~75 nm, it stays lower than 15% until the line profile approaches the big where the pore is present. The second grain is on the border with a pore, which is confirmed by the average higher N percentage (~20%) after 100 nm, than before that (~15%). Even though the difference is subtle, the qualitative behavior can still be observed. The third line profile presents similar Al and N content oscillations as the previous lines, with a higher N percentage around the pores and a lower concentration close to the Al crystals.

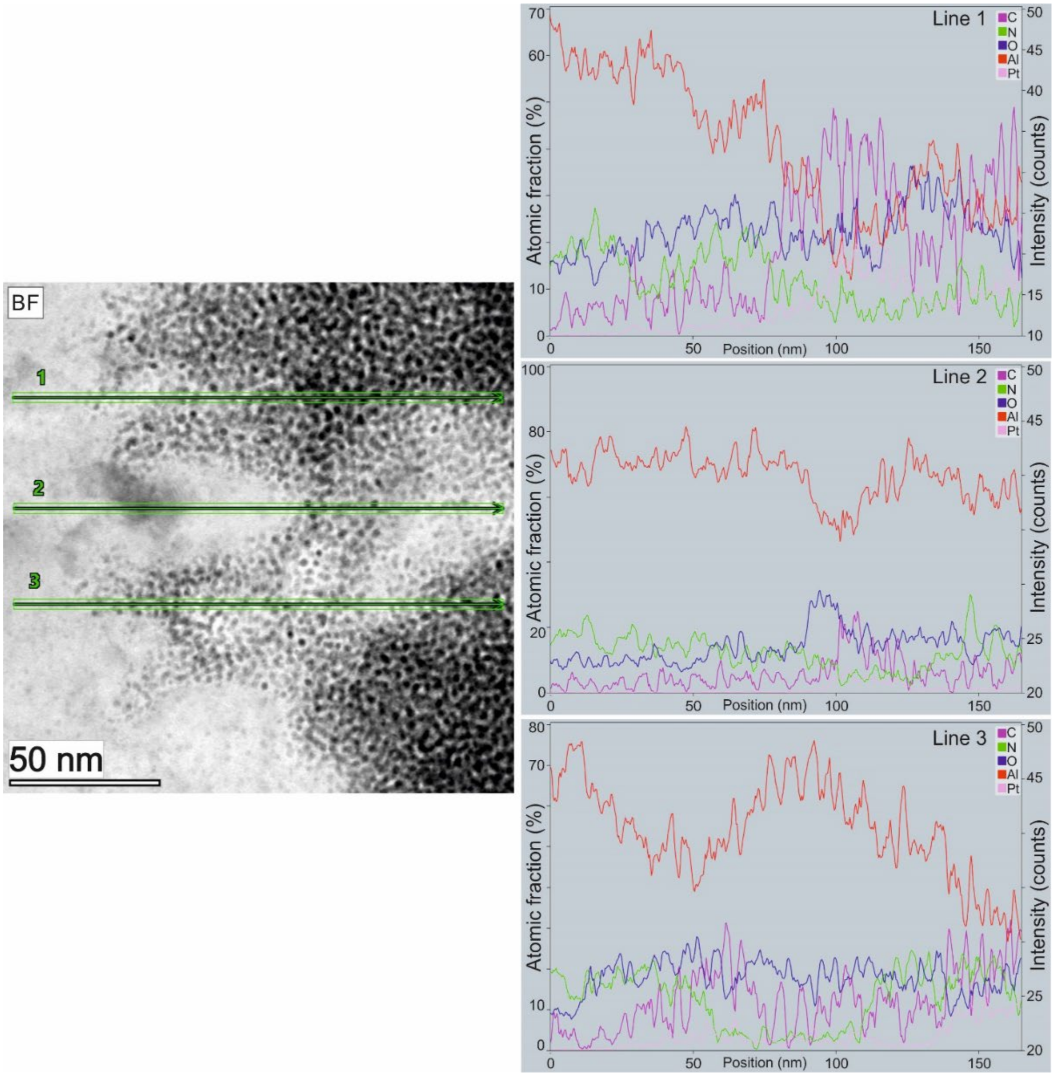

Figure A5: Line profiles of one region of the 250 nm thick B-Al thin film, shown in Figure 4 in the main manuscript.

grain on the right side of the figure (~140 nm), where the N content in the pore around the grain is higher. Otherwise, the N percentage is lower than 15%, where the Pt layer diffused within the pores of the film.

The oxygen concentration detected in the film comes from the Al oxidation under the air during the lamella preparation and the time the lamella was stored under ambient conditions. The second line profile crosses a big Al grain, followed by a small pore around 100 nm, with the N content increasing exactly

References

1 M. Klinger., CrysTBox - Crystallographic Toolbox (version 1.09) Institute of Physics of the Czech Academy of Sciences 2015.
2 M. E. Straumanis, *Journal of Applied Physics*, 1959, **30**, 1965–1969.
3 I. Levin, NIST Inorganic Crystal Structure Database (ICSD), https://data.nist.gov/od/id/mds2-2147, (accessed 4 January 2024).
